# Overexpression of Cotton a *DTX/MATE* Gene Enhances Drought, Salt, and Cold Stress Tolerance in Transgenic Arabidopsis

**DOI:** 10.3389/fpls.2019.00299

**Published:** 2019-03-12

**Authors:** Pu Lu, Richard Odongo Magwanga, Joy Nyangasi Kirungu, Yangguang Hu, Qi Dong, Xiaoyan Cai, Zhongli Zhou, Xingxing Wang, Zhenmei Zhang, Yuqing Hou, Kunbo Wang, Fang Liu

**Affiliations:** ^1^Research Base in Anyang, State Key Laboratory of Cotton Biology, Institute of Cotton Research, Chinese Academy of Agricultural Sciences (ICR, CAAS), Anyang, China; ^2^School of Physical and Biological Sciences (SPBS), Jaramogi Oginga Odinga University of Science and Technology (JOOUST), Bondo, Kenya

**Keywords:** abiotic stress, *DTX/MATE* genes, phytohormones, ABA signaling cascade, transgenic plants

## Abstract

Abiotic stresses have negative effects on plants growth and development. Plants, being sessile, have developed specific adaptive strategies that allow them to rapidly detect and respond to abiotic stress factors. The detoxification efflux carriers (DTX)/multidrug and toxic compound extrusion (MATE) transporters are of significance in the translocation of abscisic acid (ABA), a phytohormone with profound role in plants under various abiotic stress conditions. The ABA signaling cascades are the core regulators of abiotic stress responses in plants, triggering major changes in gene expression and adaptive physiological responses. We therefore carried out genome-wide analysis of the *DTX/MATE* gene family, transformed a *DTX/MATE* gene in Arabidopsis and carried out functional analysis under drought, salt, and cold stress conditions. We identified 128, 70, and 72 *DTX/MATE* genes in *Gossypium hirsutum, Gossypium arboreum*, and *Gossypium raimondii*, respectively. The proteins encoded by the *DTX/MATE* genes showed varied physiochemical properties but they all were hydrophobic. The *Gh_D06G0281* (*DTX/MATE*) over-expressing Arabidopsis lines were highly tolerant under drought, salt, and cold stress with high production of antioxidant enzymes and significantly reduced levels of oxidants. Lipid peroxidation, as measured by the level of malondialdehyde concentrations was relatively low in transgenic lines compared to wild types, an indication of reduced oxidative stress levels in the transgenic plants. Based on physiological measurements, the transgenic plants exhibited significantly higher relative leaf water content, reduced excised leaf water loss and a significant reduction in ion leakage as a measure of the cell membrane stability compared to the wild types. Abiotic stress responsive genes, *ABF4, CBL1, SOS1*, and *RD29B* were highly expressed in the transgenic lines compared to the non-transformed wild type plants. The protein encoded by the *Gh_D06G0281* (*DTX/MATE*) gene was predicted to be located within the plasma membrane. Since signals from extracellular stimuli are transmitted through the plasma membrane most of which are conducted by plasma membrane proteins it is possible the *Gh_D06G0281* (*DTX/MATE*) gene product could be important for this process.

## Introduction

Abiotic stresses such as drought, salinity, heat, cold among others continue to compromise plant growth and crop yield among the agricultural crops (Change and Production, 2010). The effects caused by the combined effect of major abiotic stresses is estimated to be over 50% loss of agricultural crops and the problem is projected to be worse as a result of environmental degradation that has continued to cause erratic weather patterns (Mittler, 2006). Plants being sessile are affected by environmental stress factors more than animals. Plants have evolved complex survival strategies to enhance their tolerance levels to both abiotic and biotic stress factors (Nakashima and Yamaguchi-Shinozaki, 2006). One of the key survival mechanisms, is the expression of various transcription factors with profound effects on minimizing the deleterious effects caused by various environmental stresses (Magwanga et al., 2018). The expression of various stress related genes has been postulated as one of the diverse mechanisms adopted by plants to enhance their adaptive response to stress factors (Bharti et al., 2016). Expression levels of certain genes has been shown to increase in response to various stress factors such as salinity, cold, drought and oxidative stress (Fang et al., 2015). The molecular mechanisms involved in drought, salt, and cold stress acclimation have been extensively investigated, and several molecules have been identified that are important for enhancing tolerance to these abiotic stresses (Huang et al., 2012). Salt stress in particular has a significant impact on gene expression (Kilian et al., 2007). Salinity-inducible genes are believed to either function as protectants or by promoting the synthesis of the osmoprotectants, detoxification enzymes, ion channels, and transporters (Rabbani, 2003).

The plant metabolites have been linked to protective role to either abiotic and or biotic stress factors, for they act as signal elicitors (Ramakrishna and Ravishankar, 2011). The synthesis of the various plant secondary metabolites has been associated with survival strategies, the amount of secondary metabolites produced are often low, thus elicitation has been widely used to accelerate the production or to induce *de novo* production of secondary metabolites in plant cell cultures (Narayani and Srivastava, 2017). The production of secondary plant metabolites is strongly dependent on the plant growth conditions and have an impact on the various plant metabolic pathways responsible for the accumulation of the related natural products, which are integral of determining the quality of the plant products (Bartwal et al., 2013). Exposure to water deficit or salinity causes many common reactions in plants. Both stresses lead to cellular dehydration, which causes osmotic stress and removal of water from the cytoplasm to vacuoles (Kissoudis et al., 2014). One of the most important secondary metabolites is abscisic acid (ABA), a plant phytohormone, which plays significant roles in various aspects of plant growth and development, including response to abiotic stresses (Vishwakarma et al., 2017). When plants are exposed to drought, salt, and cold stress conditions, ABA level increases rapidly, leading to stomatal closure and overexpression of various stress transcription factors (TFs) to cope with the stress incidence (Roychoudhury et al., 2013). The site for the synthesis of ABA is believed to be in the root tissues and therefore, it requires an elaborate transportation system to ABA target organs (Wilkinson and Davies, 2002). Proteins found to be involved in the translocation of ABA within the plant are in the multidrug and toxic compound extrusion (MATE) transporter family, which is a secondary transporter family, with the ability to translocate substrates across the membrane (Takanashi et al., 2014).

The MATE transporters are widely distributed in plants, mammals, bacteria and fungi (Chen et al., 2015). Several studies were carried out to identify and classify MATE proteins, for instance, 203 proteins have been sequenced in the MATE family (Hvorup et al., 2003), similarly 861 MATE proteins have also been identified in prokaryotes and eukaryotes (Omote et al., 2006). To date, 56 detoxification efflux carriers/multidrug and toxic compound extrusion (DTX/MATE) proteins have been identified in *Arabidopsis thaliana* (Li et al., 2002), 53 in *Oryza sativa* (Tiwari et al., 2014), and 40 in *Medicago truncatula* (Zhao and Dixon, 2009), but no work has been reported in cotton so far. Cotton is an indispensible source of raw materials for the textile industries, its production has been on the decline due to the effects of various abiotic stress factors (Zhou et al., 2014). Drought, salt, and cold stresses are the major abiotic stresses that prevent increased production in cotton, therefore in this research work, we undertook detailed analysis of the cotton *DTX/MATE* genes and transformed a novel *Gossypium hirsutum DTX/MATE* candidate gene, *Gh_D06G0281* (DTX/MATE) into the model plant *A. thaliana*, and carried out the functional characterization and expression analysis under drought, cold, and salt stress condition. The results showed that the gene has profound role in enhancing drought, cold, and salt stress factors and therefore, can be utilized in breeding of more tolerant cotton genotypes.

## Materials and Methods

### Identification, Sequence Analysis, Phylogenetic Tree Analysis and Subcellular Location Prediction of the DTX/MATE Proteins in Cotton

*Gossypium hirsutum* (AD_1_), tetraploid (AD) genome DTX/MATE protein sequences were obtained from the Cotton Research Institute website^1^. The diploid cotton *Gossypium arboreum* (A_2_) DTX/MATE protein sequences were downloaded from the Beijing Genome Institute database^2^ and *Gossypium raimondii* (D_5_) obtained from Phytozome 12 website^3^. The conserved domain of DTX/MATE protein (PF01554) was retrieved from Pfam protein family data base^4^. The hidden Markov model analysis (HMM) profile of DTX/MATE protein was queried to carry out the HMMER search^5^ against *G. hirsutum, G. raimondii*, and *G. arboreum* protein sequences. The amino acid sequences were analyzed for the presence of the DTX/MATE protein domains by use of two online tools, the ScanProsite tool^6^ and SMART program^7^. The three cotton DTX/MATE proteins, together with the DTX/MATE proteins obtained from *A. thaliana* downloaded from TAIR^8^, *O. sativa* DTX/MATE protein sequences obtained from http://rice.plantbiology.msu.edu/index.shtml were used to construct the phylogenetic tree. The multiple sequence alignments of all the DTX/MATE proteins were done by Clustal omega, MEGA 7.0 software, using an algorithm with 1000 bootstrap iterations, based on p-distance model, using complete deletion of site coverage for gaps and missing data (Magwanga et al., 2018). The physiochemical characteristics of all the obtained DTX/MATE proteins were determined by ExPASy Server tool^9^. In addition, subcellular location prediction done by WoLF PSORT^10^ and validated by TargetP1.1 Server^11^ and Protein Prowler Subcellular Localization Predictor version 1.2^12^.

### Chromosome Location and Structure Analysis of the *DTX/MATE* Genes of Upland Cotton, *G. hirsutum*

The chromosome positions of all the *DTX/MATE* genes as retrieved for the upland cotton, *G. hirsutum* (AD_1_) were obtained through a blast search of their respective gene identities in Cotton Functional Genome Database^13^. Using the coding sequences (CDS) and the genomic sequences of the *DTX/MATE* genes obtained for upland cotton, *G. hirsutum* (AD_1_), the various gene structures were analyzed in order to determine whether the gene structures were disrupted by introns or were intronless. We analyzed the gene structure in relation to intron–exon relationship. The coding and genomic sequences were analyzed through an online tool, gene structure displayer^14^.

### Analysis of the Expression Patterns of Upland Cotton *DTX/MATE* Genes in Different Tissues, Under Drought, Salt, and Cold Stress Factors Through RNA Sequencing Data

All of the *DTX/MATE* genes exhibited differential expression patterns under salt, drought, and cold stress conditions. In the transformation, a suitable gene or gene sets have to be used; therefore we obtained the RNA sequestration data from the Cotton Functional Genome Database^13^. Moreover, the main target was the abiotic stress, we analyzed the RNA sequence data in order to determine the best sets of genes with the highest expression levels across the three stress factors.

### Plant Transformation and Screening of *Gh_D06G0281* (*DTX/MATE*) Gene in Model Plant *A. thaliana* (Ecotype Colombia-0) Lines

A novel gene was obtained from upland cotton, *G. hirsutum*, which was then transformed into *A. thaliana*, ecotype Colombia-0 (Col-0). The upland cotton, *G. hirsutum* (AD_1_), accession number CRI-12 (G09091801-2) was used to confirm the expression of the *Gh_D06G0281* gene in various tissues. Upland cotton, *G. hirsutum* CRI-12, is the main upland cotton variety grown in China, due to its high yield and relatively superior fiber quality. The CRI-12 was developed by the Institute of Cotton Research, Chinese Academy of Agricultural Sciences, ICR-CAAS, thus the code ICR-12. The pWM101-35S: Gh_D06G0281 (DTX/MATE) construct in *Agrobacterium tumefaciens* GV3101 was confirmed by polymerase chain reaction (PCR) analysis with gene specific primers, the forward primer sequence *Gh_D06G0281* (*DTX/MATE*) (5′CGGATCCATGGATGGTGCCCATCGG3′) and reverse primer sequence pair of *Gh_D06G0281* (*DTX/MATE*) (5′GGTCGACTCATTTCGCCCACCTTTTAAC3′), synthesized from Invitrogen, Beijing, China. The wild type *A. thaliana* plants were transformed by the floral dip method with modifications (Clough and Bent, 1998). Infiltration media used was composed of Silwet-77 200 μl/l (0.02%), Murashige and Skoog (MS) medium 4.3 g/l, 2-(4-morpholino) ethane sulfonic acid (MES) 0.5 g/l, sucrose 50 g/l (5%), 6-benzylaminopurine (6-BA) 0.01 mg/l with pH of 5.7. Transformed lines of *A. thaliana* were selected by germinating the seeds in 50% (0.5) MS (PhytoTechnology Laboratories, Lenexa, KS, United States), containing 50 mg/l hygromycin B (Roche Diagnostics GmbH, Mannheim, Germany) for three (3) days at temperature of 4°C to optimize germination. After which, the seedlings were transferred to a growth room set at 16 h light and 8 h dark with temperature set at 25°C. After 7 days in the selection medium, at three leaf stages, the seedlings were transplanted into small plastic containers filled with vermiculite and humus in the ratio of 1:1. The seedlings at generation T_0_ were grown to set seeds, the seeds obtained were the first generation (T_1_). The T_1_ seeds were germinated in selective antibiotic medium; the one-copy lines were identified by determining the segregation ratio of 3:1 of the antibiotics-selectable marker. The 3:1 segregated lines (T_2_) seeds were again germinated in antibiotics-containing medium, only the lines with 100% were selected for the development of T_3_ generation. The T_3_ homozygous progeny was bred from a T_2_ generation after real-time quantitative reverse transcription polymerase chain reaction (RT-qPCR) and the selection of three out of the eight successfully transformed DTX/MATE over-expressing lines (OE-2, OE-3, and OE-9) was done by using *Gh_D06G0281* (*DTX/MATE*) forward primer sequence (5′ATGGTGTAGAAGGAAAGG3′) and *Gh_D06G0281* (*DTX/MATE*) reverse primer sequence (5′CGACTGATGATTGAAGGT3′) with total complementary DNA (cDNA) as the template for RT-qPCR. The phenotypic investigations were carried out in T_3_ homozygous generation.

### Determination of the Subcellular Localization of *Gh_D06G0281* (*DTX/MATE*) Protein

The open reading frame of *Gh_D06G0281* (*DTX/MATE*) was amplified by polymerase chain reaction using the transformed gene specific primer. The forward primer sequence *Gh_D06G0281* (*DTX/MATE*) 5′ACACGGGGGACTCTAGA**GGATCC**ATGGATGGTGCCCATCGGAA3′ and reverse primer sequence *Gh_D06G0281* (*DTX/MATE*) 5′ACTCATACTAGTCCCGG**GGATCC**TTTCGCCCACCTTTTAACTC3′ was synthesized by Invitrogen, Beijing, China, and the *Pfu* DNA polymerase was obtained from the TransGen Biotech, Beijing, China. The PCR products were then transformed into a plasmid, pBI121-GFP vector upstream of the green fluorescent protein (GFP) to give pBI121-*Gh_D06G0281* (*DTX/MATE*)–GFP construct with *Gh_D06G0281* (*DTX/MATE*)*–GFP* fusion gene under the regulation of CaMV 35S promoter. The cloning process was done using the pEASY-Uni Seamless Cloning and Assembly Kit, which was obtained from the TransGen Biotech, Beijing, China (Benoit et al., 2016). The construct was then transferred into *A. tumefaciens* strain LBA4404, which was obtained from the Shanghai Weidi Biotechnology Co., Shanghai, China, and transformed into epidermal cells isolated from the onion bulb. Using the method of Sun et al. (2007). The transformed onion epidermal cells were cultured in 50% Murashige and Skoog (MS) media in a dark growth chamber for 20 h at 25°C. The expression of the gene in the onion epidermal cells was observed using a Zeiss Model Axio Imager M1 Upright Fluorescent Microscope (430004-9901-Axio Imager.M1,Gottingen, Germany).

### Drought, Cold, and Salt Tolerance Determination in the Transgenic Lines

Seeds from the homozygous T_3_ generation DTX/MATE over-expressing Arabidopsis lines, OE-2, OE-3, OE-9 and the wild type seeds were sterilized by immersing in 10% bleach solutions (*v*/*v*) for 10 min and then rinsed three times with sterilized deionised water. The sterilized seeds were then sown on half–strength MS media and, after stratification in a dark chamber at 4°C for 3 days, they were incubated in a growth room at 22°C with a 16 h light/8 h dark photoperiod. After 7 days, seedlings were then transplanted into small pots filled with vermiculite and humus mixed in the ratio of 1:1. After 21 days of growth, the plants were subjected to various abiotic stress treatment; the pots were subsequently watered every 4 days with water containing 0 mM NaCl and 250 mM NaCl for 8 and 12 days. For drought stress, water was withheld for 8 and 12 days. While for cold stress, the transgenic lines and wild type were subjected to a low temperature of -10°C for 3 h then transferred to 4°C for 4 h. The chlorophyll content determination was carried out after 8 days, while observation of the phenotypic traits was done at 12 days. Three biological replicates and three technical replications were performed for all the measurements.

### Germination Rate Quantification and Root Elongation Assays

In order to determine the germination rate of the transgenic lines and the wild type under drought, salt and ABA simulated stress conditions, the overexpressing lines, OE-2, OE-3, OE-9 and the wild type seeds were sterilized and stratified, and sown in plates with 0.5 MS supplemented with varying concentrations of 0, 100, 200, and 300 mM of mannitol to simulate drought condition; exogenous ABA concentrations of 0, 0.5, 1, and 2 μM. And finally in salt stress, by supplementing the 0.5 MS with 0, 100, 150, and 200 mM of NaCl, in all the three stress levels. Germination rate was determined after 10 days. For the root length assay, the seeds of the transgenic and the wild type were sown in 0.5 MS media for 6 days then transferred to 0.5 MS supplemented with varying concentrations of 0, 100, 200, and 300 mM of mannitol to simulate drought condition; exogenous ABA concentrations of 0, 0.5, 1, and 2 μM. And finally in salt stress, by supplementing the 0.5 MS with 0, 100, 150, and 200 mM of NaCl, in all the three stress levels. The seedlings were grown for 6 days and root measurements done on the 7th day post treatment. Three biological replicates and three technical replications were performed for all the measurements.

### Measurements of Physiological Traits CMS, RLWC, and ELWL

The physiological traits such as cell membrane stability (CMS), relative leaf water content (RLWC), and Excised Leaf Water Loss (ELWL) were evaluated among the transgenic lines (OE-2, OE-3, and OE-9) and the wild type under drought, cold, and salt stress conditions. The CMS was determined as outlined by Fokar et al. (1998). The RLWC determination was done as outlined by Barrs and Weatherley (1962). Lastly ELWL, was carried out as outlined by McCaig and Romagosa (1989). Three biological replicates and three technical replicates were performed for all the measurements.

### Superoxide Dismutase (SOD) and Catalase (CAT) Enzymes Extraction and Assay

A total of 0.5 g of leaf samples were obtained from the wild type and the transgenic plants, each was ground in 5 ml of extraction buffer containing 50 mM K-phosphate buffer with a pH of 7.6 and 0.1 mM Na_2_EDTA (Amresco, Dallas, TX, United States). The mixture was then centrifuged at 15,000 rpm for 15 min. The enzymes were assayed through supernatant fractionation. All steps in the preparation of enzyme extracts were performed at 4°C. Catalase (CAT) activity was determined by monitoring the reducing level of hydrogen peroxide (H_2_O_2_), as described by Cakmak and Marschner (1992). For the determination of superoxide dismutase (SOD), the leaf samples were crushed into a fine powder on ice by the use of a mortar and pestle in 10 ml of homogenizing solution containing 50 mmol/l HEPES buffer and 0.1 mmol/l Na_2_EDTA (pH 7.6). The mixture was then centrifuged at 12,000 rpm for 20 min at 4°C. The supernatant was used for SOD assays (Yu and Rengel, 1999). The SOD activity was evaluated by monitoring the inhibition of the photochemical reduction of nitro blue tetrazolium (NBT), as described by Giannopolitis and Ries (1977) though with slight modifications. For the estimation of total SOD, a 5 ml reaction mixture was prepared with 0.025% (*w*/*v*) Triton X-100, 75 μmol/l NBT, 0.1 mmol/l EDTA, 50 mmol/l Na_2_CO_3_ (pH 10.4), 50 mM of HEPES (pH 7.6), 13 mmol/l methionine, 2 μmol/l riboflavin, and an aliquot of enzyme extract. The mixture was then illuminated with a light intensity of 350 μmol/m^2^/s for 10 min. A control reaction was set throughout with same the treatment except that the crude enzyme was replaced with an equal volume of phosphate buffer (pH 7.8) (Sahoo et al., 2001). A unit of SOD activity was then determined as the amount of enzyme required to cause 50% inhibition of the reduction of NBT at light wavelength of 560 nm (Kakkar et al., 1984). Three biological replicates and three technical replicates were performed for all the measurements.

### Chlorophyll and Malondialdehyde (MDA) Content Determination

The chlorophyll content was evaluated as per the formula described by Zhao et al. (2016). Leaf samples weighing 200 mg were obtained from the transgenic Arabidopsis lines and the wild types under stress and control conditions, and immediately frozen in liquid nitrogen before chlorophyll content was determined. Each sample was placed in 5 ml of absolute ethanol (99.9%) and heated in a water bath at 80°C for 20 min. Total chlorophyll was then evaluated in the alcohol extracts from absorbance readings, using the appropriate extinction coefficient. Chlorophyll content (mg/g fresh weight) was calculated as 100 × *A*_654_/(39.8 × sample fresh weight) as described by Tetley and Thimann (1974). For the determination of MDA the lipid peroxidation was measured as the amount of MDA quantified by the thiobarbituric acid (TBA) reaction (Heath and Packer, 1968). The leaf samples were ground in two volumes of ice-cold 0.1% (*w*/*v*) trichloroacetic acid (TCA) and then centrifuged for 15 min at 15,000 rpm. The mixture containing 1 ml of the supernatant and 2 ml of 0.5% (*w*/*v*) TBA in 20% (*w*/*v*) TCA was then heated at a temperature of 95°C for 30 min and then immediately cooled in an ice cold bath. The cooled mixture was then centrifuged at 12,000 rpm for 10 min at 4°C. The supernatant absorbance was then read at light wavelength of 532 nm and the values corresponding to non-specific absorption recorded at a light wavelength of 600 nm.

### RT-qPCR Analysis of the Expression of Stress-Responsive Genes in Transgenic Arabidopsis

We carried out the analysis of the expression of *Gh_D06G0281* (*DTX/MATE*) and the stress-responsive genes in the transgenic Arabidopsis lines. The RNA was isolated from 1 month-old transgenic seedlings (OE-2, OE-3, and OE-9) and their wild types (Col-0 ecotype) grown under normal conditions (CK) and the three stress conditions salt, drought, and cold stresses. Under cold stress, the transgenic lines and the wild types were exposed to extremely low temperature at -10°C for 3 h, then transferred to 4°C for 4 h, similar method has also been used in screening for cold stress tolerance in transgenic rice (Yoon et al., 2016). For salt stress tolerance, the wild type and the transgenic lines were grown in 250 mM NaCl concentration for 4 days, while under drought condition, water was totally withdrawn for 4 days. RNA extraction was then done by use of EASYspin Plus plant RNA extraction kit, obtained from Aid Lab, was used as outlined in the instruction manual. The concentration and quality of the extracted RNA samples were evaluated through gel electrophoresis and a NanoDrop 2000 spectrophotometer. The extracted RNAs were reverse-transcribed into cDNAs before being used as templates in RT-qPCR analysis. Expression of Gh_D06G0281 (DTX/MATE) and the stress-responsive genes in the DTX/MATE over-expressing lines, OE-2,OE-3, OE9 Arabidopsis transgenic plants was performed through RT-qPCR analysis using gene-specific primers (Table 1), using the FastaStart Universal SYBR-Green Master in a detection system, obtained from the Roche Diagnostics GmbH, Mannheim, Germany. Arabidopsis *actin2* gene, *Atactin2*-Forward sequence (5′TTGTGCTGGATTCTGGTGATGG3′) and *Atactin2*-Reverse sequence (5′CCGCTCTGCTG TTGTGGTG3′) was used as the control in the RT-qPCR reactions. The RT-qPCR mixture preparation was done in accordance with the manufacturer’s instructions. The RT-qPCR reaction mixture was prepared as previously outlined by Magwanga et al. (2018) in the analysis of *LEA* genes in cotton. The RT-qPCR analysis was done in triplicate for all the samples, each representing biological trials.

**Table 1:** Identification of the DTX/MATE genes in cotton genomes.

Gene name	Gene description	*G. hirsutum*	*G. arboreum*	*G. raimondii*
*DTX1*	Protein DETOXIFICATION 1	0	1	0
*DTX2*	Protein DETOXIFICATION 2	0	1	0
*DTX3*	Protein DETOXIFICATION 3	0	0	1
*DTX6*	Protein DETOXIFICATION 6	1	0	1
*DTX7*	Protein DETOXIFICATION 7	0	1	0
*DTX8*	Protein DETOXIFICATION 8	2	1	2
*DTX9*	Protein DETOXIFICATION 9	1	1	0
*DTX11*	Protein DETOXIFICATION 11	1	0	0
*DTX12*	Protein DETOXIFICATION 12	4	3	2
*DTX14*	Protein DETOXIFICATION 14	4	1	2
*DTX15*	Protein DETOXIFICATION 15	1	1	1
*DTX16*	Protein DETOXIFICATION 16	6	3	2
*DTX18*	Protein DETOXIFICATION 18	4	3	6
*DTX19*	Protein DETOXIFICATION 19	4	3	3
*DTX21*	Protein DETOXIFICATION 21	4	3	3
*DTX24*	Protein DETOXIFICATION 24	3	1	1
*DTX25*	Protein DETOXIFICATION 25	2	3	3
*DTX27*	Protein DETOXIFICATION 27	8	4	4
*DTX29*	Protein DETOXIFICATION 29	4	2	2
*DTX33*	Protein DETOXIFICATION 33	4	2	2
*DTX34*	Protein DETOXIFICATION 34	2	1	1
*DTX35*	Protein DETOXIFICATION 35	2	1	1
*DTX37*	Protein DETOXIFICATION 37	1	1	1
*DTX40*	Protein DETOXIFICATION 40	5	2	2
*DTX41*	Protein DETOXIFICATION 41	4	2	2
*DTX42*	Protein DETOXIFICATION 42	4	2	2
*DTX43*	Protein DETOXIFICATION 43	4	2	2
*DTX44*	Protein DETOXIFICATION 44	4	1	3
*DTX45*	Protein DETOXIFICATION 45	2	1	1
*DTX46*	Protein DETOXIFICATION 46	5	3	3
*DTX48*	Protein DETOXIFICATION 48	6	3	2
*DTX49*	Protein DETOXIFICATION 49	11	5	5
*DTX51*	Protein DETOXIFICATION 51	10	4	5
*DTX53*	Protein DETOXIFICATION 53	6	3	3
*DTX54*	Protein DETOXIFICATION 54	2	1	1
*DTX55*	Protein DETOXIFICATION 55	4	2	2
*DTX56*	Protein DETOXIFICATION 56	3	2	1

**Totals**		**128**	**70**	**72**

### RNA Isolation and RT-qPCR Analysis of the Transformed Gene in Upland Cotton, *G. hirsutum* Tissues

The EASYspin Plus plant RNA extraction kit was obtained from Aid Lab, was used to extract RNA of the plant samples under normal conditions and under salt, drought, and cold stress treatments. Under normal conditions, RNA was extracted from the roots, stem, sepal, leaf, petal, stamen, pistil, seed, and fiber tissues in three biological replicates; this was to determine which tissue exhibited higher up-regulation of the transformed gene. In salt, drought, and cold stress conditions leaf, root and stem tissues were collected for RNA extraction at 0, 3, 6, 12, and 24 h of salt, drought, and cold stress exposure. The quality and concentration of each RNA samples were determined as outlined in Section “RT-qPCR Analysis of the Expression of Stress-Responsive Genes in Transgenic Arabidopsis” above. The Actin7 gene forward sequence 5′ATCCTCCGTCTTGACCT TG3′ and reverse sequence 5′TGTCCGTCAGGCAACTCAT3′ applied as the reference gene together with the transformed gene, *Gh_D06G0281* (*DTX/MATE*) gene primer forward sequence 5′ATGGTGTAGAAGGAAAGG3′ and reverse sequence 5′CGACTGATGATTGAAGGT′ was used for RT-qPCR analysis.

## Results

### Identification, Sequence, and Structure Analysis of DTX/MATE Candidate Proteins in Cotton

We identified 128, 72 and 70 genes that encode the DTX/MATE proteins in *G. hirsutum, G. raimondii*, and *G. arboreum*, respectively (Table 2). The number of *DTX/MATE* genes varied across the three cotton genomes. There were 11 DTX/MATE genes of the DTX49 sub-type in *G. hirsutum* and 5 in *G. arboreum*. The DTX18 subgroup of genes was most abundant in with six genes. Our analysis of the physiochemical properties of the putative proteins encoded by the *DTX/MATE* genes across the three cotton genomes showed that, protein lengths (aa) ranged from 55 to 868 aa. The widest range in protein length was observed in *G. hirsutum*, while in *G arboreum*, the lengths of the putative DTX/MATE proteins ranged from 84 to 722 aa, while in the lengths of putative DTX/MATE proteins *G. raimondii* ranged from 133 aa to 601 aa. The molecular weight (MW) of the various DTX/MATE proteins exhibited a wider range among the three cotton genomes, with the minimum molecular weight of 5.73 kDa as detected among the DTX/MATE proteins in *G. hirsutum* (Gh_Sca216225G01.1). The highest molecular weight value was 94.36 kDa (*Gh_D13G1550.1*) (Supplementary Table S1). Similar results have also been recorded for the analysis of the proteins encoded by the *DTX/MATE* genes in *Populus trichocarpa*, in which their molecular masses ranged from 13.14 to 65.49 kDa (Li et al., 2017). The Isoelectric point (pl) values ranged from 4.288 to 10.046 and Grand Average of Hydropathy (GRAVY) values ranged from 0.315 to 1.113, indicating that the proteins encoded by the DTX/MATE proteins are generally hydrophobic, indicating that they may be membrane proteins (Huang et al., 2014). Hydrophobicity is a characteristic trait of various proteins encoded by the stress inductive genes, such as the late embryogenesis abundant (*LEA*) genes (Magwanga et al., 2018). In relation to the structures of the *DTX/MATE* genes, majority of the genes were interrupted by introns except 29 genes in *G. hirsutum*, 11 genes in *G. raimondii* and 16 in *G. arboreum* (Supplementary Table S1).

**Table 2:** The stress specific gene primers used in RT-qPCR analysis for the expression of the salt, drought, and cold stress tolerance in the transgenic lines.

Gene	Forward sequence	Reverse sequence
*AtRD29B*	5′-CCAGATAGCGGAGGGGAAAGGACAT-3′	5′-AAGTTCACAAACAGAGGCATCATCATCATAC-3′
*AtSOS1*	5′-TCGTTTCAGCCAAATCAGAAAGT-3′	5′-TTTGCCTTGTGCTGCTTTCC-3′
*AtABF4*	5′-AACAACTTAGGAGGTGGTGGTCAT-3′	5′-TGTAGCAGCTGGCGCAGAAGTCAT-3′
*AtCBL1*	5′-GAAATGAAACTGGCTGATGAAACCATAGAG-3′	5′-CTCGTGGCAATCTACTCGGTCTTAAACC-3′

### Phylogenetic Analysis and Functional Classification of DTX/MATE Candidate Proteins in Cotton

To characterize the phylogenetic relationships among the cotton DTX/MATE proteins, full protein sequences of 128, 70, and 72 DTX/MATE proteins from *G. hirsutum, G. arboreum*, and *G. raimondii*, respectively, together with the DTX/MATE protein sequences from *O. sativa* (56) and *A. thaliana* (57) were used to construct a phylogenetic tree through multiple sequence alignments using ClustalW in MEGA 7.0. The putative DTX/MATE proteins from cotton and other plants were grouped into three sub families designated as D1, D2, and D3 with six smaller sub groups. Sub family D1 with 230 DTX/MATE proteins was the largest group, while subfamily D2, which includes 99 DTX/MATE proteins and subfamily D3, which has 54 DTX/MATE proteins, were smaller D1, D2, and D3 were further subdivided into sub groups designated as D1–1, D1–2 for D1, D2–1 and D2–2 for D2 and finally, the D3 sub family was classified into D3–1 and D3–2. Across the entire phylogenetic tree, no orthologous gene pairs were detected between cotton to any other plants used, all the orthologous gene pairs were formed among the cotton genomes, for instance *Gorai.009G232700* and *Gh_D05G2137* (Supplementary Figure S1). The cloned gene was found to be in D1, sub group D1–1, and is to Gorai.010G037100, being that they were in the same clade, but the other members of the sub group included 3 genes from *O. sativa*, 3 genes from *A. thaliana*, 4 from *G. raimondii*, 4 from *G. arboreum* and 8 genes from *G. hirsutum* (Supplementary Figure S1). The results from our phylogenetic tree analysis were consistent with the previous results obtained for the genome wide analysis of the DTX/MATE proteins in *Populus*, in which all the DTX/MATE proteins were sub divided into three main groups (Li et al., 2017).

### Chromosome Mapping and Subcellular Localization of the DTX/MATE Candidate Proteins in Cotton

All the 128 genes were mapped across the 26 linkage groups of the *G. hirsutum*, except for 4 genes, *Gh_Sca216225G01* (*DTX51*), *Gh_Sca150596G01* (*DTX49*), *Gh_Sca011468G01* (*DTX41*), and *Gh_Sca004831G01* (*DTX48*), which were not annotated and thus classified as scaffold. The distribution of genes within the sub genomes was consistent, with 63 and 61 genes being mapped in At and Dt sub genomes, respectively. However, there was evidence for gene loss between the two sets of homeologous chromosomes. For example, chrA02 has a single *DTX/MATE* gene, while its homeolog, chromosome D02 had 8 genes. Similarly, chromosome A03 has 12 *DTX/MATE* genes while chromosome D03 only has 2. In the A genome, 68 genes were mapped in all the 13 chromosomes; only 2 genes were grouped as scaffold. The highest density of gene loci was observed on chr11 with 13 genes while the fewest were seen in chr02, with only 2 genes mapped. Lastly, in the D genome, 71 *DTX/MATE* genes were distributed across the 13 chromosomes, with only one being mapped into the scaffold region. The most gene loci were detected on chr09 with 13 genes accounting for 21% of all the *DTX/MATE* genes in the D genome. Chr12 (1 gene), chr03 (2 genes), chr01 (3 genes), and chr10 (3 genes) were found to harbor the fewest *DTX/MATE* genes (Supplementary Figure S2). The *DTX/MATE* genes tend to cluster on either the upper or lower arms of the chromosomes, for instance chromosome A03 with 8 genes clustered on the lower arm, and in chromosome A11, six genes were found to be located on the upper arm. The loci detection on the upper or lower arms, has also been observed among stress responsive genes such as *LEA* genes (Magwanga et al., 2018).

The proteins encoded by *DTX/MATE* genes were found to be located in eight (8) different cellular compartments. The highest proportions of DTX/MATE proteins was predicted to be located in the plasma membrane, with 234 (87%) of DTX/MATE proteins encoded by genes in all three cotton genomes. In addition, 19 of these proteins were predicted to be vacuolar, 7 nuclear and 7 chloroplast localized. Only single DTX/MATE proteins were predicted to be located in the cytosol, endoplasmic reticulum (E.R) and extracellular spaces (Supplementary Table S2). These results obtained for the sub cellular localization prediction was in agreement with the previous reports, in which 82.91% of the DTX/MATE proteins in soybean were found to be localized within the plasma membrane (Liu et al., 2016).

**FIGURE 1:**
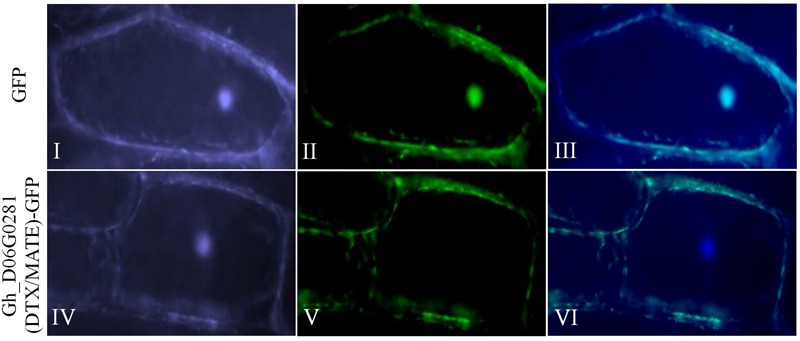
Localization of *Gh_D06G0281* (*DTX/MATE*) in onion epidermal cells. **(I–III)** Onion epidermal cells transformed with 35S::GFP; **(IV–VI)** onion epidermal cells transformed with 35S: Gh_D06G0281_*GFP* **(I,IV)**: light field with magnification of 400× to display morphology. **(II,V)** Dark field images for the detection of green fluorescent protein (GFP) fluorescence. **(III,VI)** Superimposed light and dark field images.

Based on the bioinformatics analysis, an online tool WoLF PSORT sub cellular localization tools showed that the protein encoded by *Gh_D06G0281* (*DTX/MATE*) was predicted to be located within the plasma membrane. We sought to determine if the protein encoded by the transformed gene was embedded within the plasma membrane or not. We therefore made a 35S: DTX/MATE:GFP (*Gh_D06G0281-*GFP) fusion vector pB1121-*Gh_D06G0281* (*DTX/MATE*) which was delivered into the onion epidermal cells by particle bombardment (Abdollahi et al., 2009). The positive control showed that the protein encoded by the gene was located at the plasma membrane (Figure 1).

### RNA Sequence and RT-qPCR Analysis of the Native *Gh_D06G0281* (*DTX/MATE*) Gene in Upland Cotton Tissues

**FIGURE 2:**
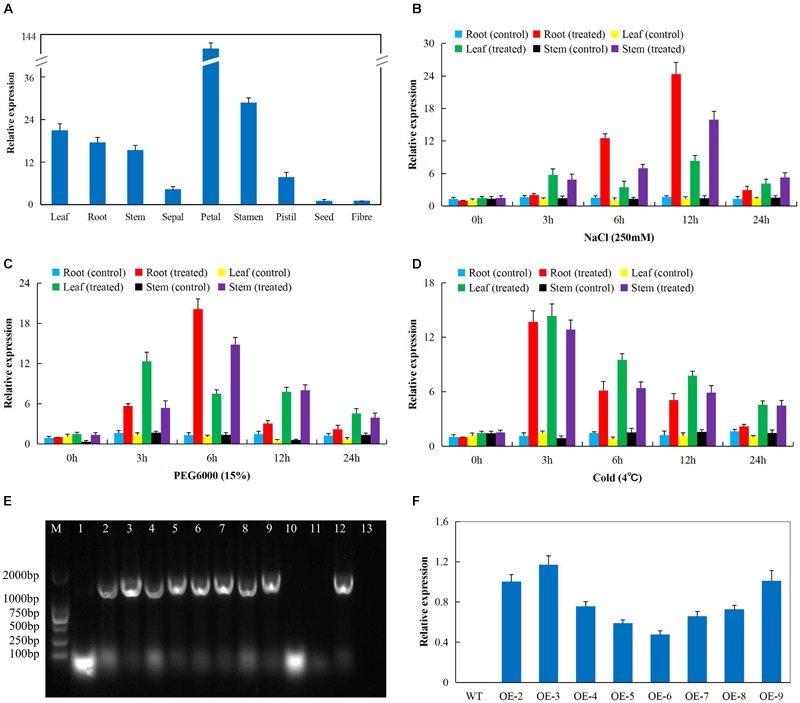
The RT-qPCR analysis of the expression of the cloned gene *Gh_D06G0281* (*DTX/MATE*) **(A)** Total RNA isolated from various tissue of cotton plant under normal conditions. **(B)** Total RNA extracted from salt-stressed cotton seedlings. **(C)** Total RNA extracted from drought-stressed cotton seedlings. **(D)** Total RNA extracted from cold-stressed cotton seedlings. **(E)** Polymerase chain reaction (PCR) analysis performed to check 1479 bp coding sequence (CDS) integration in transformed T_1_ generation, number 1–11 transgenic lines, 12 positive control (*pWM101-Gh_D06G0281* (*DTX/MATE*) and 13 is the negative control (wild type). **(F)** The transcripts expression levels of the *Gh_D06G0281* (*DTX/MATE*) of T_2_ transgenic lines analyzed through RT-qPCR, in three biological replicates.

In order to determine the best gene to be transformed into the model plant, *A. thaliana* (Col-0 ecotype); we obtained RNA sequence data from the cotton functional genome database^15^. The RNA sequence data obtained for drought, salt, and cold stress conditions showed that *Gh_D06G0281* (*DTX/MATE*) was highly expressed under the three abiotic conditions (Supplementary Table S3). We went further to validate the RNA sequence data through RT-qPCR analysis in upland cotton, *G. hirsutum* on the root, stem and leaf tissues under drought, salt, and cold stress conditions. The gene was abundantly expressed in the reproductive tissues, more specifically in the petal and stamen (Figure 2A). In addition, we carried out expression analysis of the *Gh_D06G0281* (*DTX/MATE*) gene in cotton seedlings under salt (250 mM NaCl), cold (4°C) and osmotic stress (PEG6000_15%) conditions, and compared the expression level of the gene in the three tissues both under treatment and no treatment controlled condition. The three tissues used for expression analysis, root, stem, and leaf tissues were obtained at 0, 3, 6, 12, and 24 h interval after stress treatment. The gene exhibited differential expression in the various tissues examined under stress and normal conditions and showed significantly higher expression levels in all the three tissues examined under stress conditions. Under salinity condition, *Gh_D06G0281* (*DTX/MATE*) was highly up regulated in the root tissues at 6 and 12 h (Figure 2B). Under osmotic stress, the root tissue showed higher up regulation of *Gh_D06G0281* (*DTX/MATE*) compared to the other two tissues, leaf and stem (Figure 2C). Deviation occurred under cold stress, the leaves showed higher induction of *Gh_D06G0281* (*DTX/MATE*) gene across the time series (Figure 2D,E).

### Analysis of Physiological Traits in the Transgenic Lines and Wild Type Under Salt, Drought, and Cold Stress Conditions

**FIGURE 3:**
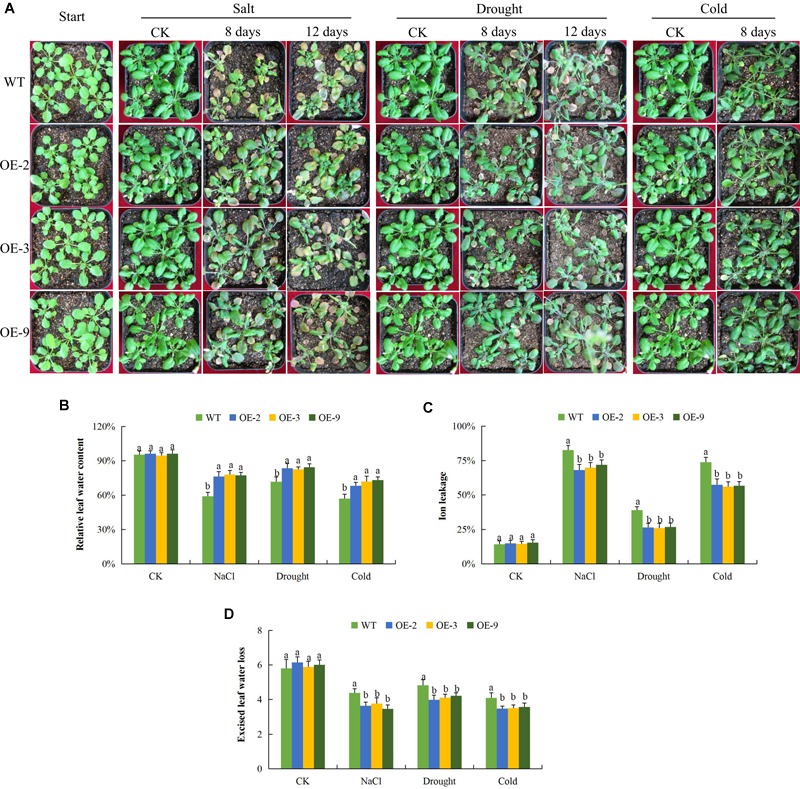
Physiological traits measurements in *Gh_D06G0281* (*DTX/MATE*) transgenic lines under salt, drought and cold stress conditions. **(A)** Transgenic lines and wild type under abiotic stress conditions **(B)**. Quantitative determination of relative water content (RLWC). **(C)** Quantitative determination of cell membrane stability (CMS) as ion leakage concentration. **(D)** Quantitative determination of excised leaf water loss (ELWL) in leaves of wild type and both DTX/MATE over-expressing (OE) lines (OE-2, OE-3, and OE-9) after 8-day post stress exposure. In **(B–D)**, each experiment was repeated three times. Bar indicates standard error (SE). Different letters indicate significant differences between wild type and OE lines (ANOVA; *p* < 0.05). CK: normal conditions.

The physiological measurements associated with abiotic stress tolerances include RLWC, CMS as measured by the ion leakage concentration and ELWL (ElBasyoni et al., 2017). We tested the three transgenic lines and wild type in order to evaluate their physiological responses toward salt, drought, and cold stress conditions. There was no significant difference in all the measurements among the transgenic lines and wild type under controlled conditions but when the plants were exposed to various stress conditions, the transgenic lines exhibited increased stress tolerance as compared to the wild type (Figure 3). RLWC, was significantly higher among the transgenic lines, than in the wild type; this was evident under salt, osmotic stress and cold stress conditions (Figure 3B). Wild type plants exhibited higher levels of stress-induced ion leakage compared to the plants of the transgenic lines (Figure 3C). Lastly, we evaluated the rate of water loss between the leaves of wild type and transgenic plants, the ELWL was not significantly different under controlled condition among plants of the wild type and transgenic lines, but under stress conditions, the wild type leaves exhibited a higher level of water loss as compared to the leaves obtained from the transgenic plants (Figure 3D). The plants with enhanced tolerance to various abiotic stresses such as drought, salt, and cold stresses were found to have relatively lower levels of ion leakage and higher RLWC (Blum and Ebercon, 1981). The increased level of tolerance among the three transgenic lines showed that the transformed gene had a positive effect in enhancing salt, drought, and cold stress tolerance in the transgenic Arabidopsis lines.

### Analysis of Oxidant and Antioxidant Enzyme Content in the Transgenic Lines and Wild Type Under Drought, Cold, and Salt Stress Conditions

**FIGURE 4:**
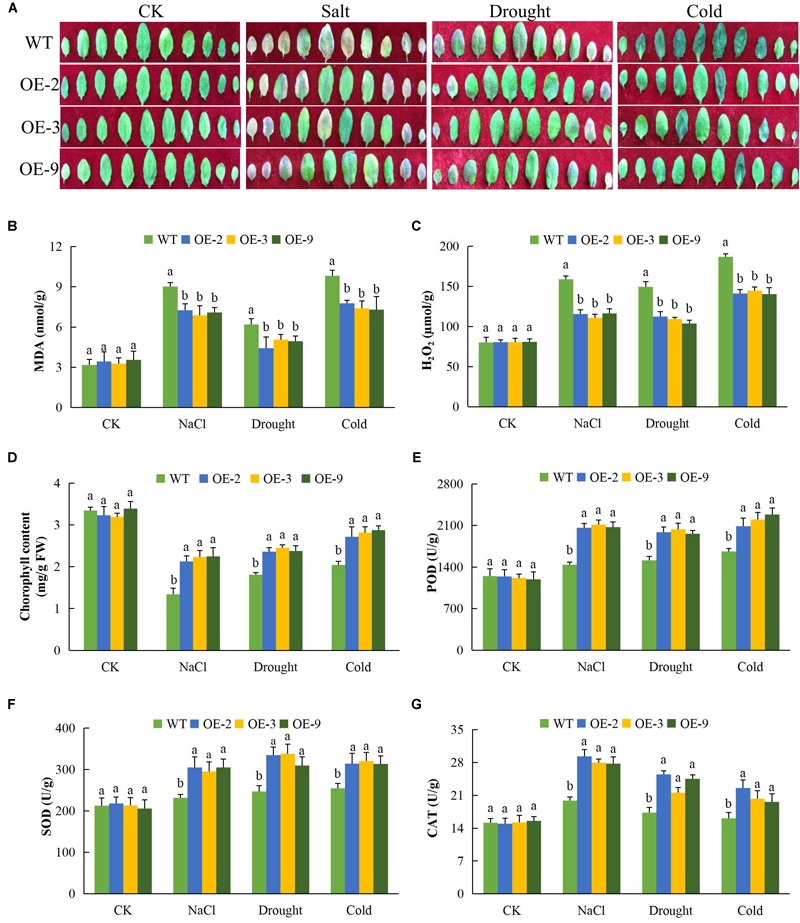
Alteration of leaf morphology, determination of oxidants and antioxidant enzymes concentration levels in *Gh_D06G0281* (*DTX/MATE*) transgenic lines under salt, drought, and cold stress conditions. **(A)** Leaf size and pattern of each rosette leaf beginning from the oldest one of transgenic lines and wild types. The plants were grown for 8 days under 16 h light and 8 h dark photoperiod before photographs **(B)**. Quantitative determination of malondialdehyde (MDA) concentration. **(C)** Quantitative determination of hydrogen peroxide (H_2_O_2_) concentration. **(D)** Quantitative determination of chlorophyll content. **(E)** Quantitative determination of POD concentration. **(F)** Quantitative determination of SOD concentration. **(G)** Quantitative determination of CAT in leaves of wild type and both DTX/MATE over-expressing (OE) lines (OE-2, OE-3, and OE-9) after 8-day post stress exposure. In **(B–G)**, each experiment was repeated three times. Bar indicates standard error (SE). Different letters indicate significant differences between wild type and OE lines (ANOVA; *p* < 0.05). CK: normal conditions.

Exposure of plants to salt, drought, and cold stresses, along with other forms of stresses, do lead to the excess production of reactive oxygen species (ROS) in plants which are highly toxic to the cell (Miller et al., 2010). The released ROS has deleterious effects on the proteins, lipids, carbohydrates, and DNA which ultimately results in oxidative stress (Schieber and Chandel, 2014). Overproduction of ROS species triggers defensive antioxidant systems to protect plants against oxidative stress damage (Birben et al., 2012). In this research work, we determined the levels of various oxidants and antioxidant enzyme activities in the transgenic and wild type plants under salt, drought, and cold stress conditions (Figure 4). While leaf size was reduced in wild type plants under osmotic stress, salt and cold stresses, leaves of DTX/MATE over-expressing lines retained their size and were not disfigured due to stress exposure (Figure 4A). Since levels of lipid peroxidation can be used to evaluate the deleterious effects associated with excessive production of ROS, we assayed the concentration of MDA in leaves of transgenic and wild type plants. Significantly lower levels of MDA were seen in leaves of the transgenic lines compared to wild types (Figure 4B). Similarly, H_2_O_2_ levels increased in stressed leaves from wild type plants but these increase were significantly lower in leaves from transgenic lines (Figure 4C). The data showed that overexpression of DTX/MATE plays a role in minimizing the effects of oxidative stress by regulating the amount of ROS.

We also determined the activity of levels of the antioxidant enzymes peroxidase (POD), SOD and CAT, along with chlorophyll content. There was no significant difference between the transgenic lines and the wild type under normal conditions, but under salt, cold, and osmotic stress conditions, there was a more substantial significant reduction in the wild type plants than in transgenic DTX/MATE over-expressing plants. For example under salt stress, the chlorophyll content in the wild type was below 2 mg/gFW while the transgenic lines were all above 2 (Figure 4D). The same trend was observed under drought and cold stress conditions. The activities of POD (Figure 4E), SOD (Figure 4F), and CAT (Figure 4G); were significantly higher in transgenic plants than in wild type plants.

### Overexpression of *Gh_D06G0281* (*DTX/MATE*) in Plants Confers ABA Hypersensitivity for Seed Germination and Root Elongation

**FIGURE 5:**
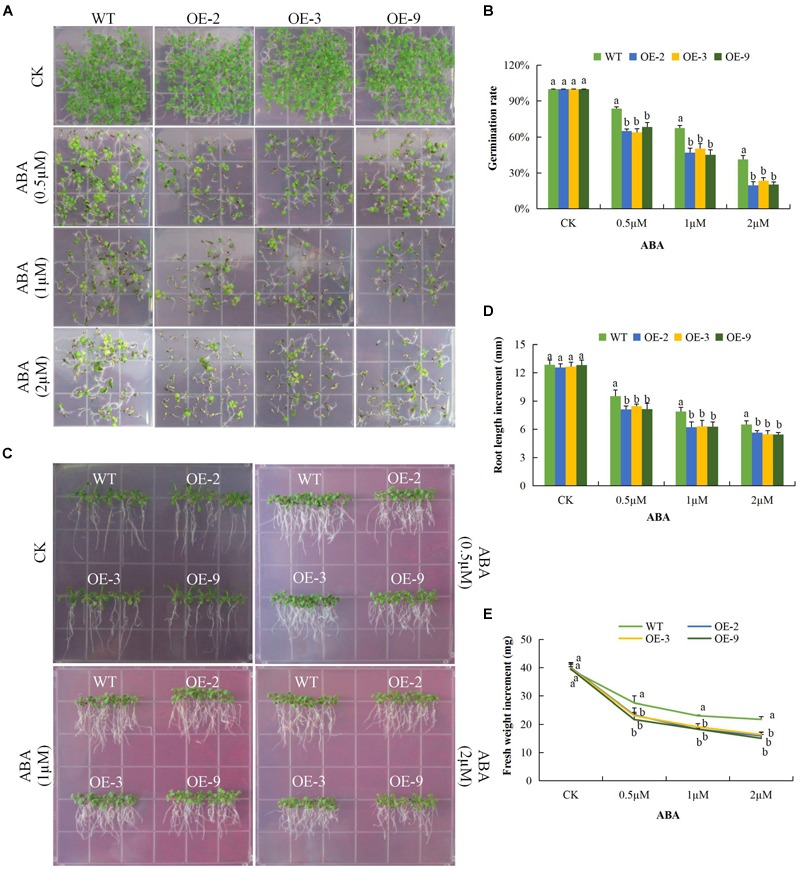
Overexpression of *Gh_D06G0281* (*DTX/MATE*) causes hypersensitivity to ABA-elicited seed germination, root growth inhibition and fresh biomass accumulation in. transgenic plants and WT. **(A)** Seed germination rate of transgenic lines and wild type on ABA 0.5 MS with (0, 0.5, 1, and 2 μM) for 10 days. **(B)** Quantitative comparisons of germination rate of the transgenic and wild type on ABA 0.5 MS with (0, 0.5, 1, and 2 μM) after 10 days. **(C)** Root elongation comparisons on 0.5 MS with varied ABA concentrations 0, 0.5, 1, and 2 μM. The seedlings were scored and photographed after 6 days post germination. **(D)** Quantitative determination of root lengths. **(E)** Quantitative determination of fresh weight biomass of wild type and both DTX/MATE over-expressing (OE) lines (OE-2, OE-3, and OE-9) after 6 days post germination in different ABA concentrations. In **(B,D,E)**, each experiment was repeated three times. Bar indicates standard error (SE). Different letters indicate significant differences between wild type and OE lines (ANOVA; *p* < 0.05). CK: normal conditions.

In order to determine the response of seeds of DTX/MATE over-expressing lines to ABA, OE-2, OE-3, and OE-9 seeds, along with wild types, were tested for their ability to germinate when treated with various concentrations of ABA. While at 0 μM ABA differences in germination rate were insignificant, the rate of germination in wild type was inversely proportional to ABA concentration (Figure 5A,B), germination of seeds from the transgenic lines was reduced more substantially and these differences were statistically significant. We further investigated the effect of ABA on root growth; the wild type less inhibition of root growth compared to the DTX/MATE over-expressing lines, OE-2, OE-3, and OE-9, across the varying ABA concentrations (Figure 5C,D). Finally, we evaluated the whole plant biomass as a measure of total fresh weight of the entire plants after 6 days growth in 0.5 MS media infused with different ABA concentrations (Figure 5C,E) under ABA treatment, the transformed plants had produced less biomass compared to the wild type. The negative effects of exogenous ABA on seed germination, root growth, and biomass accumulation in DTX/MATE over-expressing plants, relative to wild type, indicates that these plants are more sensitive to ABA.

### Overexpression of *Gh_D06G0281* (*DTX/MATE*) Gene in Plants Confers Tolerance to Drought, Salt, and Cold Stress Tolerance

**FIGURE 6:**
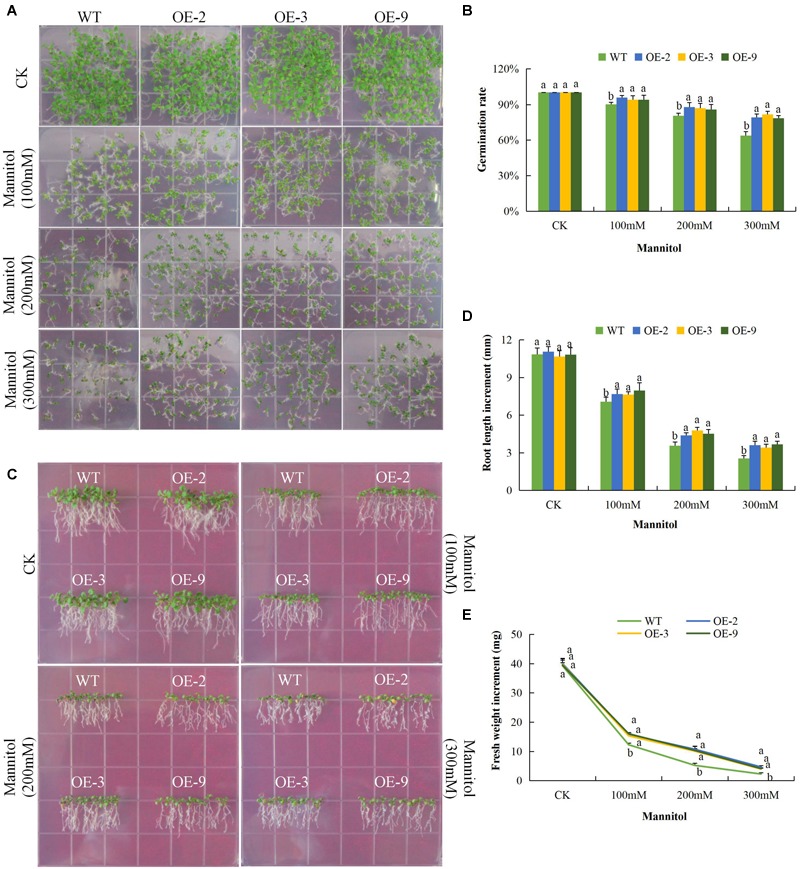
Overexpression of *Gh_D06G0281* (*DTX/MATE*) effects on germination, root growth and fresh biomass accumulation in transgenic plants and wild type under osmotic stress. **(A)** Seed germination rate of transgenic lines and wild type on mannitol 0.5 MS with (0, 100, 200, and 300 mM) for 10 days. **(B)** Quantitative comparisons of germination rate of the transgenic and WT on mannitol 0.5 MS with (0, 100, 200, and 300 mM) for 10 days. **(C)** Root elongation comparisons on mannitol 0.5 MS with (0, 100, 200, and 300 mM). The seedlings were scored and photographed after 6 days post germination. **(D)** Quantitative determination of root lengths. **(E)** Quantitative determination of fresh weight biomass of wild type and both DTX/MATE over-expressing (OE) lines (OE-2, OE-3, and OE-9) after 6 days post germination in different mannitol concentrations. In **(B,D,E)**, each experiment was repeated three times. Bar indicates standard error (SE). Different letters indicate significant differences between wild type and OE lines (ANOVA; *p* < 0.05). CK: normal conditions.

We investigated the response of DTX/MATE over-expressing and wild type seeds and seedlings to drought, salt and cold stress conditions in relation to seed germination rate, primary root length elongation and fresh biomass accumulation. The DTX/MATE over-expressing lines showed enhanced performance with increased seed germination rate, relatively increased primary root growth and with higher fresh biomass accumulation compared to the wild type in drought, salt, and cold stress conditions. A steady decline in the rate of germination was seen with increased mannitol concentration (Figure 6A,B). The DTX/MATE over-expressing lines had significantly longer roots compared to the wild type under mannitol treatment (Figure 6C,D), indicating increased tolerance to tolerance to osmotic stress. The DTX/MATE over-expressing lines also accumulated more biomass compared to the wild type under mannitol treatment (Figure 6C,E).

**FIGURE 7:**
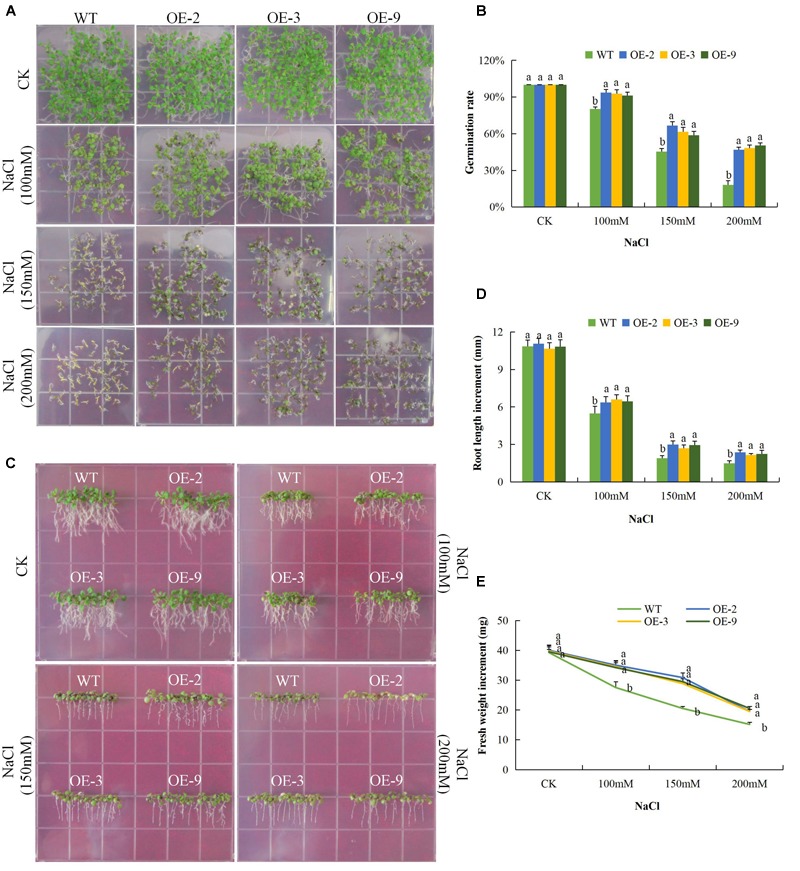
Overexpression of *Gh_D06G0281* (*DTX/MATE*) enhances salt stress tolerance. **(A)** Seed germination rate of transgenic lines and wild type in 0.5 MS with supplemented with (0, 100, 150, and 200 mM) NaCl for 10 days. **(B)** Quantitative comparisons of germination rate of the transgenic and wild type on 0.5 MS with supplemented with (0, 100, 150, and 200 mM) NaCl for 10 days. **(C)** Root elongation comparisons on 0.5 MS with supplemented with (0, 100, 150, and 200 mM) NaCl for 10 days. The seedlings were scored and photographed after 10 days post germination. **(D)** Quantitative determination of root lengths. **(E)** Quantitative determination of fresh weight biomass of wild type and both DTX/MATE over-expressing (OE) lines (OE-2, OE-3, and OE-9) after 6 days post germination in different NaCl concentrations. In **(B,D,E)**, each experiment was repeated three times. Bar indicates standard error (SE). Different letters indicate significant differences between wild type and OE lines (ANOVA; *p* < 0.05). CK: Normal conditions.

**FIGURE 8:**
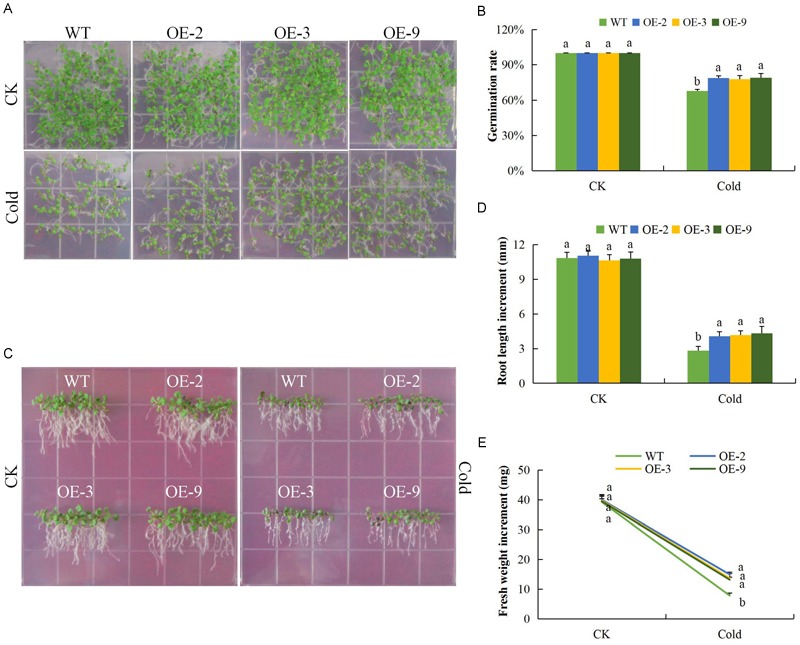
Overexpression of *Gh_D06G0281* (*DTX/MATE*) enhances cold stress tolerance. **(A)** Seed germination rate of transgenic lines and wild type on 0.5 MS put at normal and low temperature (4°C) for 10 days. **(B)** Quantitative comparisons of germination rate of the transgenic and wild type on 0.5 MS put at normal and low temperature (4°C) for 10 days. **(C)** Root elongation comparisons on 0.5 MS put at normal and low temperature (4°C) for 6 days. The seedlings were scored and photographed after 6 days post germination. **(D)** Quantitative determination of root lengths. **(E)** Quantitative determination of fresh weight biomass of wild type and both DTX/MATE over-expressing (OE) lines (OE-2, OE-3, and OE-9) after 6 days post germination at normal and low temperature of 4°C. In **(B,D,E)**, each experiment was repeated three times. Bar indicates standard error (SE). Different letters indicate significant differences between wild type and OE lines (ANOVA; *p* < 0.05). CK: normal conditions.

Under salinity stress, the germination rate of both wild type and DTX/MATE over-expressing lines were significantly reduced and the inhibition increased with increasing salt concentration. The percent germination was estimated to be below 30% in wild type but remained much higher in DTX/MATE over-expressing lines (Figure 7A,B). Root growth was negatively in the wild seedlings, while the transgenic lines maintain a significantly higher root elongation under salt stress condition (Figure 7C,D). The over-expressing lines had significantly higher fresh weight accumulation than wild type, although they showed a decreasing trend in biomass accumulation with increased salt concentration (Figure 7C,E). Similar performance was observed among the transgenic lines under cold stress (Figure 8A–E). When exposed to low temperatures (4°C) transgenic DTX/MATE over-expressing lines maintained higher levels of seed germination and significantly longer primary roots and greater fresh biomass accumulation than wild type (Figure 8).

### Analysis of the Abiotic StressResponsive Genes in the Transgenic andWild Type Plants

**FIGURE 9:**
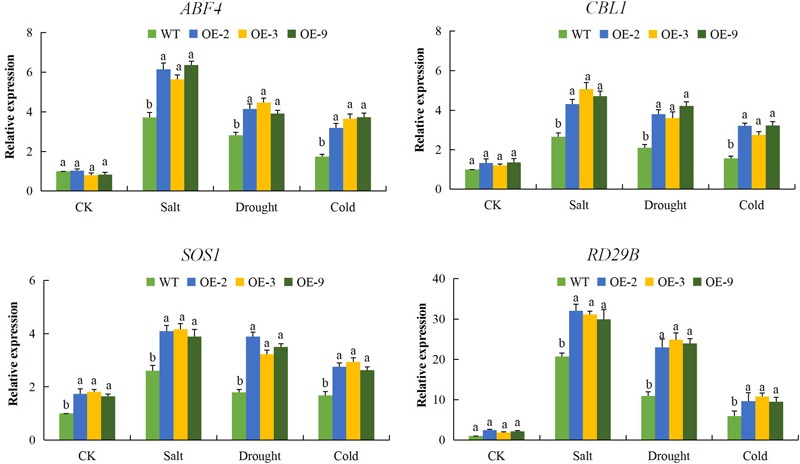
Expression levels of abiotic stress-responsive genes (*ABF4, CBL1, SOS1*, and *RD29B*) in transgenic lines (OE-2, OE-3, and OE-9) and wild type Arabidopsis *Atactin2* gene was used as the reference, each experiment was repeated three times, mean values with ± SD, (a,b) calculated by Student’s *t*-test with *p* < 0.05.

To better understand the mechanism by which DTX/MATE over-expression affects the stress tolerance phenotype of transgenic Arabidopsis plants, we compared the expression of other known abiotic stress responsive genes, including calcineurin B-like protein1 (*CBL1*), salt overly sensitive 1 (*SOS1*) and responsive to desiccation 29B (*RD29B*) and one transcription factor, ABRE-binding factor 4 (*ABF4*). These genes have been widely investigated in relation to various abiotic stress factors, the SOS signal transduction pathways, which includes SOS1, SOS2, and SOS3 are critical for maintaining ion homeostasis in salt stressed plants (Zhu, 2003). All of these abiotic stress responsive genes exhibited significant up regulation in the three DTX/MATE over-expressing Arabidopsis lines. Compared to the wild type (Figure 9), an indication that overexpression of this gene has a positive effect on stress responsive gene expression in these plants. It seems likely that these changes in gene responsiveness could play a role in increased stress tolerance seen in these plants.

## Discussion

Due to the ever changing environmental conditions, occasioned with loss of arable land to urbanization and contamination, plants are in constant exposure to extreme growing conditions, which has resulted in to massive losses in terms of yield and quality of various agricultural products (Mirzabaev, 2013). Plants, being sessile, have evolved various adaptive strategies in order to acclimatize to these extreme growing conditions. One of these adaptation is the rapid expression of various genes with profound roles in enhancing stress tolerance (Grotewold and Springer, 2017). Recent studies have shown that there are a number plant stress responsive genes with profound roles in enhancing abiotic stress tolerance in plants, such as late embryogenesis abundant (*LEA*) proteins (Magwanga et al., 2018), plant hormones such as ABA (Danquah et al., 2014) among others. In this research work, we sought to investigate the role of a recently discovered transporter, the DTX/MATE protein family, which has been found to be responsible for ABA efflux within the plants, enhancing tolerance to aluminum toxicity and other abiotic stresses (Tiwari et al., 2014). The DTX/MATE proteins family belong to the protein functional domain of PF01554, these groups of proteins do functions as secondary transporter protein (Shoji, 2014). The MATE transporters are widely distributed in plants, bacteria, fungi, and mammals (Omote et al., 2006).

Despite the wider distribution of these proteins in plants, little information is known in relation to their possible role in enhancing abiotic stress tolerance. In the analysis of the two *MATE* genes from rice, *OsMATE1* and *OsMATE2*, the transgenic lines showed early bolting, higher seeding rate, increased leaf size and possibly found to be involved in enhancing various stress tolerance (Tiwari et al., 2014). In cotton, we found 128, 70, and 72 DTX/MATE proteins in *G. hirsutum* (AD_1_), *G. arboreum* (A_2_), and *G. raimondii* (D_5_). The genes were found to be distributed in all the chromosomes, both in tetraploid and diploid cotton genomes. The abundant presence of these proteins encoded by *DTX/MATE* genes in cotton showed that these genes could be having a functional role apart from transporting substances across the membranes within the cells. The physiochemical properties of the proteins encoded by DTX*/MATE* genes showed that the proteins were varied in terms of molecular weight, protein length, isoelectric values, grand average hydropathy values, the charge and even intron–exon interruption ratios. Based on the GRAVY values the proteins had values less than 1, ranging from 0.315 to 0.934, an indication that the proteins were hydrophobic in nature, a property common among the membranous proteins (Bagag et al., 2013). The hydrophobic nature of the *DTX/MATE* genes is correlates with various stress related genes such as the *LEA* (Magwanga et al., 2018), heat shock protein chaperones such as *Hsp60, Hsp70*, and *Hsp90* which do binds with non-native proteins and thus prevent their aggregation during abiotic stress conditions (Wang et al., 2004). The cloned gene *Gh_D06G0281*, was found to be in the same sub group with genes such as *AT5G5380* (putative ripening-responsive protein) and *AT5G44050* (putative protein MATE family) which have been found to be playing an active role in the transportation of calcium ions (Ca^2+^), thereby enhancing salt stress tolerance in plants (Maathuis, 2006). The *MATE* genes have also been associated with plant growth, for instance a defect in the Arabidopsis *ALF5* gene inhibits root growth in plants, the gene has been found to be embedded in the vacuoles of the root epidermis (Diener et al., 2001), *ALF5* gene also confers tolerance to tetraethylammonium (TEA) in yeast (Diener et al., 2001). The transparent testa 12 (*tt12*) gene in Arabidopsis do encodes a MATE-type transporter (Frangne et al., 2002), which acts as a vacuolar flavonoid/H^+^ - antiporter active in proanthocyanidin-accumulating cells of the seed coat and facilitates vacuolar uptake of epicatechin 3′-*O*-glucoside for proanthocyanidin biosynthesis in *A. thaliana* and *M. truncatula* (Zhao and Dixon, 2009). The detection of these two genes, *AT5G10420* (*DTX26*) and *AT5G65380* (*DTX27*) within the same sub group with the cloned gene, indicate that the novel gene has a functional role under various abiotic stress factors in plants.

The DTX/MATE transporters primarily function as organic acid and secondary metabolite transporters, A protein encoded by DTX50 from Arabidopsis was found to function as ABA efflux transporter and plays a significant role in response to drought stress (Remy et al., 2013). Plants that over-express DTX/MATE showed significant reductions in oxidative damage under salt, cold and osmotic stress conditions that correlate with increased levels of antioxidant enzymes such as SOD, CAT, and POD. Plant chloroplasts, peroxisomes, and mitochondria are the sites of ROS production (Venditti et al., 2013). Abiotic stresses such as drought, cold, and salt stress have been found to augment the production of ROS and lead to ROS-associated injury (Giraud et al., 2008). The excess ROS produced reacts with reduced Fe^2+^ and Cu^2+^ producing a highly toxic OH, the uncharged hydroxyl radicals (^.^OH) has the ability to penetrate the cellular membranes (Rhoads et al., 2006). Peroxidation of mitochondrial membranes is initiated by the abstraction of a hydrogen atom by ROS, especially by OH. This leads to the formation of cytotoxic lipid aldehydes, alkenals, and hydroxyalkenals, such as the 4-hydroxy-2-nonenal and MDA. Once formed, the lipid peroxidation (LPO) products can cause cellular damage by reacting with proteins, nucleic acids, and lipids which can eventually result in plant death. The other site of ROS production in plants is the chloroplast, which is normally reduced under normal condition (Pfannschmidt, 2003). However, when plants are exposed to abiotic stresses such as excess light, drought, salt stress and CO_2_ limiting conditions, enhances the production of ROS in chloroplasts is increased (Asada, 2006). Analysis in plants of the DTX/MATE over-expressing the transgenic lines showed higher concentrations of antioxidant enzymes and reduced levels of oxidants compared to the wild type. The ability of the transgenic plant to induce the expression of antioxidant enzymes indicates that DTX/MATE may be involved in stress responsive signaling cascades that regulate the response of these plants to drought, cold, and salt stresses. These results are consistent with previous results with plants that express SOS1 (Chen et al., 2017).

Abiotic stress damages the selective permeability of the plasma membrane. Thus cells cannot maintain their internal environment which negatively affects plant growth and development (Munns, 2002). In order to determine if the transgene had any effect on maintaining the CMS and osmotic balance within the cell, we evaluated ion leakage percentage, RLWC and ELWL in both transgenic and wild types. The results showed that the DTX/MATE over-expressing lines (OE-2, OE-3, and OE-9) showed significantly lower levels of ion leakage under drought, salt, and cold stress conditions compared to the wild type. Similarly, the transgenic lines showed significantly higher levels of relative water content and relatively lower levels water loss from excised leaves. The results obtained were consistent with the previous findings in which transgenic tobacco plants exhibited lower levels of ion leakage, higher relative water content and significantly lower levels of leaf water loss under drought stress conditions compared to the wild type plants (Huo et al., 2016). The altered physiological responses of the transgenic lines indicates that the expression of the *DTX/MATE* gene had a significant effect on enhancing tolerance levels to salt, drought, and cold stress conditions. The expression of four known abiotic stress responsive genes, *ABF4, CBL1, SOS1*, and *RD29B* were significantly higher in all the three DTX/MATE over-expressing lines (OE-2, OE-3, and OE-9) compared to the wild type. Additional stress regulatory genes, including *CBL, RD29a*, and *ABF4* were also found to be upregulated in DTX/MATE overexpressing plants. (Liang et al., 2016). The upregulation of these abiotic stress responsive genes in the transgenic Arabidopsis plants showed that the *DTX/MATE* gene had a functional role in enhancing tolerance to drought, salinity and cold stress in the transgenic plant.

## Author Contributions

PL, RM, KW, and FL designed the experiments, implemented and collected the data. PL and RM analyzed the results and prepared the manuscript. RM, PL, JK, YaH, QD, FL, XW, XC, ZhoZ, ZheZ, and KW revised the manuscript. All authors reviewed and approved the final manuscript.

## Conflict of Interest Statement

The authors declare that the research was conducted in the absence of any commercial or financial relationships that could be construed as a potential conflict of interest.

## Footnotes

1 http://mascotton.njau.edu.cn

2 https://www.bgi.com/

3 http://www.phytozome.net/

4 http://pfam.xfam.org

5 http://hmmer.janelia.org/

6 http://prosite.expasy.org/scanprosite/

7 http://smart.embl-heidelberg.de/

8 http://www.arabidopsis.org/

9 https://web.expasy.org/compute_pi/

10 https://wolfpsort.hgc.jp/

11 http://www.cbs.dtu.dk/services/TargetP/

12 http://bioinf.scmb.uq.edu.au:8080/pprowler_webapp_1-2/

13 https://cottonfgd.org

14 http://gsds.cbi.pku.edu.cn/

15 https://cottonfgd.org/analyze/

## Supplementary Material

FIGURE S1 Phylogenetic tree analysis and multiple sequence alignment of *Gh_D06G0281* (*DTX/MATE*) protein. **(A)** *Gh_D06G0281* (*DTX/MATE*) proteins tree analysis built using MEGA 7.0 program together with proteins from other plants as illustrated in the key. **(B)** Phylogenetic tree analysis of the sub set D1 sub family in which the cloned gene belongs. **(C)** Amino acids alignment of *Gh_D06G0281* (*DTX/MATE*) with other DTX/MATEs from the three cotton genomes, *Oryza sativa* and *Arabidopsis thaliana*.

FIGURE S2 Chromosomal mapping of the MATE genes in the various cotton chromosomes. **(A)** mapping in tetraploid cotton chromosomes, *G. hirsutum*. **(B)** Mapping in diploid cotton chromosomes, *G. raimondii*. **(C)** Mapping in diploid cotton chromosomes, *G. arboreum*.

TABLE S1 Physiochemical properties of the three cotton genomes proteins encoded by the *MATE* genes.

TABLE S2 Subcellular localization prediction of the proteins encoded by the *MATE* genes in cotton.

TABLE S3 RNA expression profiling datum for the upland cotton *MATE* genes.
